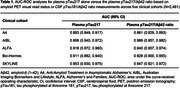# Clinical performance and effect of pre‐analytical variation of plasma pTau217 alone versus the plasma pTau217/Aβ42 ratio for the identification of amyloid pathology

**DOI:** 10.1002/alz70861_108585

**Published:** 2025-12-23

**Authors:** Christopher M. Rank, Joana Amorim Freire, Alexander Jethwa, Annunziata Di Domenico, Christina Rabe, Marc Suárez‐Calvet, Colin L. Masters, Tobias Bittner

**Affiliations:** ^1^ Roche Diagnostics GmbH, Penzberg, Bavaria Germany; ^2^ Roche Diagnostics International Ltd, Rotkreuz, Zug Switzerland; ^3^ Genentech, Inc., South San Francisco, CA USA; ^4^ Hospital del Mar Research Institute, Barcelona, Barcelona Spain; ^5^ Servei de Neurologia, Hospital del Mar, Barcelona Spain; ^6^ Barcelonaβeta Brain Research Center (BBRC), Pasqual Maragall Foundation, Barcelona Spain; ^7^ Florey Institute of Neuroscience and Mental Health, University of Melbourne, Melbourne, VIC Australia; ^8^ F. Hoffmann‐La Roche Ltd, Basel, Basel‐Stadt Switzerland

## Abstract

**Background:**

Plasma phosphorylated tau 217P (pTau217) is a promising biomarker for aiding in the detection of amyloid pathology. Studies suggest the plasma pTau217 to plasma amyloid‐β (1–42) (Aβ42) ratio may outperform pTau217 alone. This analysis compared the clinical performance of plasma pTau217 alone versus the plasma pTau217/Aβ42 ratio to identify amyloid pathology across five cohorts spanning the Alzheimer’s disease spectrum. The impact of pre‐analytical variation on biomarker levels was also evaluated.

**Method:**

Plasma pTau217 and Aβ42 levels were measured in samples from cognitively impaired/unimpaired individuals from A4, AIBL, ALFA, Bio‐Hermes, and SKYLINE studies using the prototype Elecsys^®^ Phospho‐Tau (217P) Plasma and Elecsys β‐amyloid (1–42) Plasma immunoassays (both Roche Diagnostics International Ltd, Rotkreuz, Switzerland). Assay performance was assessed based on amyloid positron emission tomography visual read status or cerebrospinal fluid (CSF) measurements of the phosphorylated tau 181P to Aβ42 ratio (CSF pTau181/Aβ42) using area under the curve‐receiver operating characteristic (AUC‐ROC) analyses. Short‐term sample stability at different storage conditions representative of routine procedures (4°C and room temperature [RT]) was assessed in independent plasma samples from healthy donors.

**Result:**

Across the five cohorts (*N* =2,481), AUC values ranged from 0.883–0.956 for plasma pTau217 alone and 0.861–0.969 for the plasma pTau217/Aβ42 ratio (Table 1). In the short‐term sample stability experiment (*N* =15), plasma pTau217 alone was highly stable in whole blood/plasma at 4°C and RT for ≤3 days, whereas the plasma pTau217/Aβ42 ratio and plasma Aβ42 alone were stable in whole blood/plasma at 4°C for ≤24 hours and RT for <6 hours showing strong bias at later time points.

**Conclusion:**

Plasma pTau217 alone accurately identified amyloid pathology across five cohorts; the addition of Aβ42 did not consistently improve performance. While plasma pTau217 alone was stable under various storage conditions, the plasma pTau217/Aβ42 ratio and plasma Aβ42 alone were less stable. This instability could lead to misinterpretation of biomarker results and pose challenges for broad implementation in routine clinical practice worldwide.